# Synthesis of [Fe(L_eq_)(L_ax_)]*_n_* coordination polymer nanoparticles using blockcopolymer micelles

**DOI:** 10.3762/bjnano.8.133

**Published:** 2017-06-26

**Authors:** Christoph Göbel, Ottokar Klimm, Florian Puchtler, Sabine Rosenfeldt, Stephan Förster, Birgit Weber

**Affiliations:** 1 Inorganic Chemistry II, University of Bayreuth, Universitätsstr. 30, 95440 Bayreuth, Germany; 2 Inorganic Chemistry I, University of Bayreuth, Universitätsstr. 30, 95440 Bayreuth, Germany; 3 Physical Chemistry I and Bavarian Polymer Institute, University of Bayreuth, Universitätsstr. 30, 95440 Bayreuth, Germany

**Keywords:** block copolymer, composite, nanoparticles, self-assembly, spin crossover

## Abstract

Spin-crossover compounds are a class of materials that can change their spin state from high spin (HS) to low spin (LS) by external stimuli such as light, pressure or temperature. Applications demand compounds with defined properties concerning the size and switchability that are maintained when the compound is integrated into composite materials. Here, we report the synthesis of [Fe(L_eq_)(L_ax_)]_n_ coordination polymer (CP) nanoparticles using self-assembled polystyrene-*block*-poly(4-vinylpyridine) (PS-*b*-P4VP) block copolymer (BCP) micelles as template. Variation of the solvent (THF and toluene) and the rigidity of the axial ligand L_ax_ (L_ax_ = 1,2-di(pyridin-4-yl)ethane) (bpea), *trans*-1,2-di(pyridin-4-yl)ethene (bpee), and 1,2-di(pyridin-4-yl)ethyne) (bpey); L_eq_ = 1,2-phenylenebis(iminomethylidyne)-bis(2,4-pentanedionato)(2−)) allowed the determination of the preconditions for the selective formation of nanoparticles. A low solubility of the CP in the used solvent and a high stability of the Fe–L bond with regard to ligand exchange are necessary for the formation of composite nanoparticles where the BCP micelle is filled with the CP, as in the case of the [FeL_eq_(bpey)]*_n_*@BCP. Otherwise, in the case of more flexible ligands or ligands that lead to high spin complexes, the formation of microcrystals next to the CP–BCP nanoparticles is observed above a certain concentration of [Fe(L_eq_)(L_ax_)]*_n_*. The core of the nanoparticles is about 45 nm in diameter due to the templating effect of the BCP micelle, independent of the used iron complex and [Fe(L_eq_)(L_ax_)]*_n_* concentration. The spin-crossover properties of the composite material are similar to those of the bulk for FeL_eq_(bpea)]*_n_*@BCP while pronounced differences are observed in the case of [FeL_eq_(bpey)]*_n_*@BCP nanoparticles.

## Introduction

Nanomaterials and especially nanocomposites of coordination polymers (CPs) and (porous) coordination networks are of great interest in current research because of their various applications as sensors, data-storage devices, catalysts or contrast agents [1–5]. For these applications the formation of stable, uniform and monodisperse particles with defined properties is necessary. Synthetic procedures for nanoparticles with size control (gold [6–7], metal oxides [8–9]) and/or shape control (gold and silver [10]) are already well known. The reduction of metal salts is very common for noble metals [11], while (fast) precipitation or inverse-micelle technique are often used for metal oxides (mostly magnetite) [12]. For coordination polymers (CP) or networks a limited amount of methods are applicable because of the very demanding reaction conditions and/or incompatible reactants. Recently we demonstrated that the use of block copolymers (BCPs) is a highly promising and easy approach for the size control of CPs [13]. BCPs form micellar structures through self-assembly in specific solvents and can therefore be used as nanoreactors [14–16]. Using this approach, a very controlled miniaturisation of coordination polymers or networks can be envisioned, provided it is easily transferable to other systems. In this work we will analyse which preconditions need to be fulfilled for a successful synthesis of uniform CP–BCP nanoparticles.

Coordination polymers with spin crossover (SCO) properties are well known in the literature [4–5,17–18], but their miniaturisation into precisely defined nanomaterials with SCO properties comparable to those of the bulk material is still in its infancy [19–23]. SCO materials can be switched by external stimuli such as temperature, pressure or light between a high spin (HS) and a low spin (LS) state [5,18]. Switching between these two states alters physical properties such as magnetism, structure or colour, which make these materials interesting for sensors [2,24–26], display devices [27–29] or as functional contrast agents [30–34]. The SCO properties deeply depend on the precise control of size and crystallinity of the nanocomposite. Most commonly the inverse-micelle technique is used for the preparation of nanoparticles [35–39]. However, the spin-crossover properties of the bulk are often lost upon miniaturisation and only few examples preserving the hysteresis (bistability) in a nanostructured system are known [21,40–43]. This is most likely due to a loss of the crystallinity of the particles. Especially SCO complexes are highly sensitive to small changes in the crystal packing and thus excellently suited to investigate the impact of nanostructuration of the material. In our recent work [13] we used the block copolymer polystyrene-*b*-poly(4-vinylpyridine) (PS-*b*-P4VP) to prepare spherical nanoparticles of the 1D spin-crossover coordination polymer [FeL_eq_(bipy)]*_n_*. We were able control the crystallinity of the [FeL_eq_(bipy)]*_n_* core through successive addition of starting material and by variation of the reaction time and temperature. Having a high crystallinity of the core, the SCO properties were closer to those of the bulk material (thermal hysteresis loop).

**Scheme 1:**
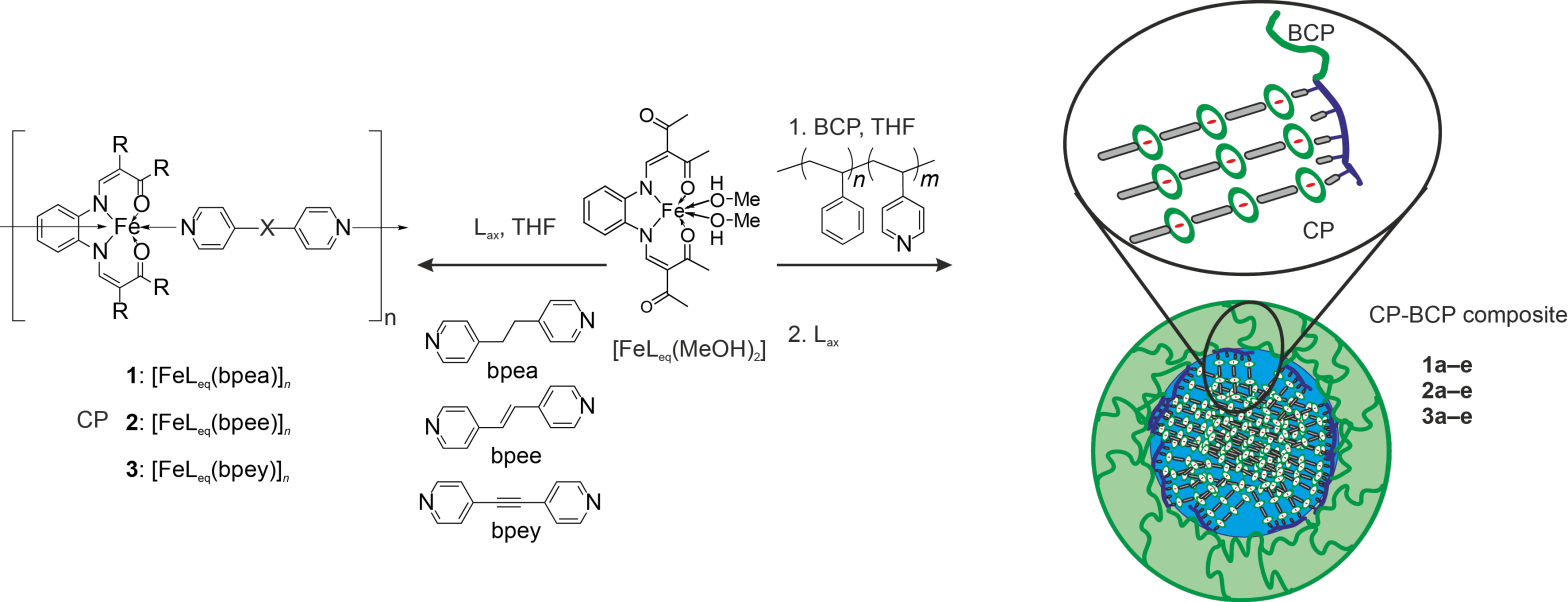
Synthesis of the three different coordination polymers [FeL_eq_(bpea)]*_n_* (**1**), [FeL_eq_(bpee)]*_n_* (**2**) and [FeL_eq_(bpey)]*_n_* (**3**) and the respective coordination polymer–block copolymer composites (CP–BCP) [FeL_eq_(bpea)]*_n_*@BCP (**1a**–**e**), [FeL_eq_(bpee)]*_n_*@BCP (**2a**–**e**) and [FeL_eq_(bpey)]*_n_*@BCP (**3a–e**).

We herein report the synthesis of three further coordination polymer block copolymer nanocomposites (CP–BCP) using the same synthesis strategy. This allows us to investigate the influence of the coordination polymer on the formation and the SCO activity of the final nanocompound. The CPs differ in the axial ligands (L_ax_), namely 1,2-di(pyridin-4-yl)ethane (bpea), *trans*-1,2-di(pyridin-4-yl)ethene (bpee) and 1,2-di(pyridin-4-yl)ethyne (bpey) (Scheme 1). The ligands were chosen because of their different flexibility. From the synthesis of the bulk complexes it is known, that an increasing flexibility of the ligand leads to an increase in solubility of the obtained CP [44–45]. This way we can investigate the impact of the solubility of the CP on the selective formation of nanoparticles in the BCP micelle cores. In Scheme 1, the general approach and the abbreviations used for the different samples are given.

## Results and Discussion

### Bulk complexes

**Figure 1:**
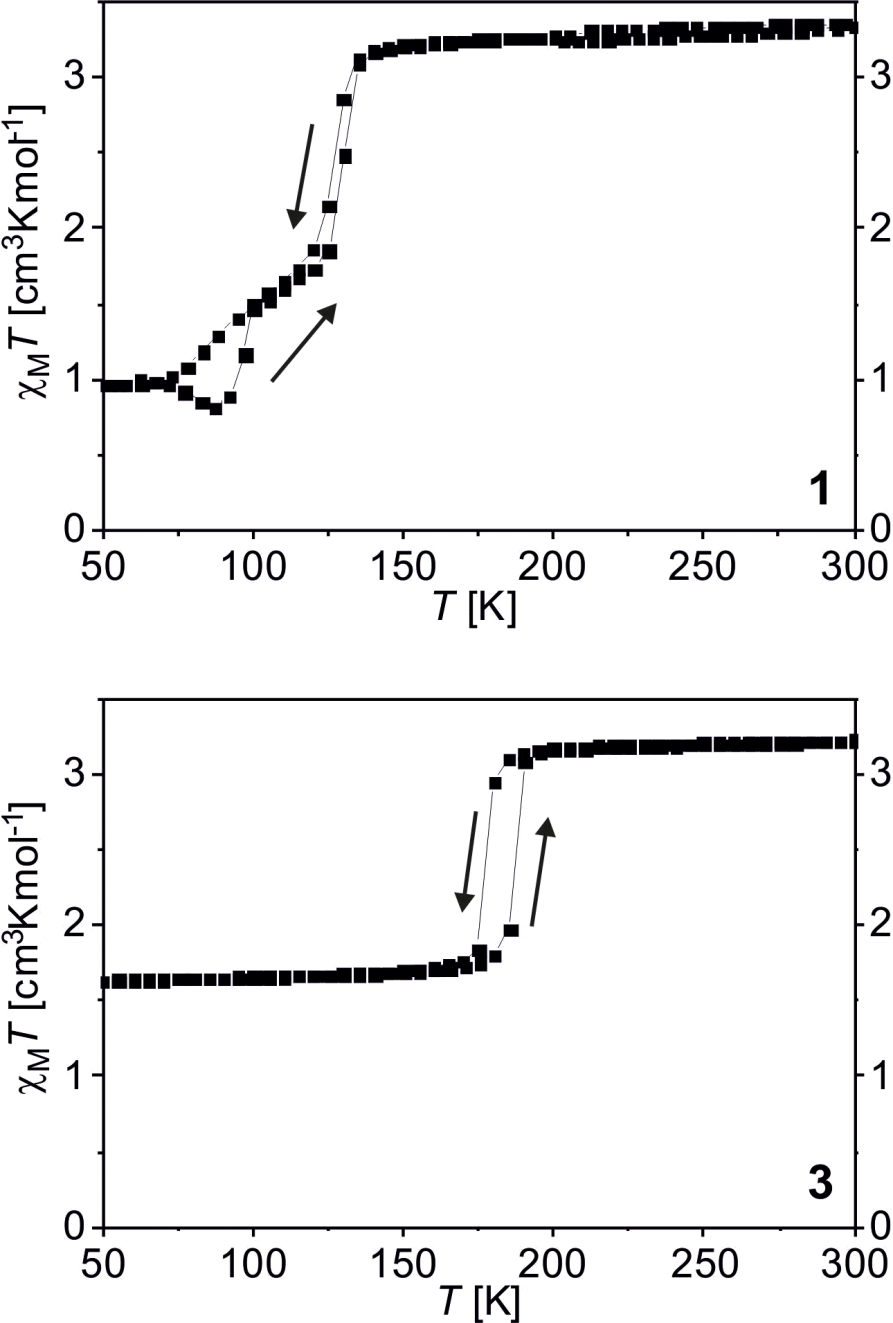
Magnetic susceptibility data for the coordination polymers [FeL_eq_(bpea)]*_n_* (**1**) and [FeL_eq_(bpey)]*_n_* (**3**), which undergo spin crossover.

The magnetic properties of SCO coordination polymers often depend on solvent molecules included in the crystal packing [46–49]. To allow a comparison between bulk material and nanoparticles and to study the influence of nanostructuring on magnetism, the bulk complexes were synthesised in THF and their magnetic properties were investigated. [FeL_eq_(bpea)]*_n_* and [FeL_eq_(bpee)]*_n_* were already synthesised in methanol [44,50], the coordination polymer [FeL_eq_(bpey)]*_n_* is described here for the first time. The coordination polymers **1**, **2** and **3** were synthesised by dissolving the iron(II) complex [FeL_eq_(MeOH)_2_] and the respective axial ligand in THF. The solution was refluxed for 1 h. After cooling down overnight, the fine crystalline precipitate was filtered off and dried in vacuo to yield brown or dark violet powders. The crystals were too small for single-crystal X-ray structure analysis. In Figure 1, the magnetic properties of [FeL_eq_(bpea)]*_n_* (**1**) and [FeL_eq_(bpey)]*_n_* (**3**) as plot of the χ_M_*T* product (χ_M_ = magnetic susceptibility, *T* = temperature) as a function of the temperature is given. Sample **1** is paramagnetic at RT with a χ_M_*T* value of 3.25 cm^3^·K·mol^−1^, typical for iron(II) in the HS state [51]. Upon cooling the χ_M_*T* value remains constant down to 140 K where an abrupt, incomplete spin crossover occurs. In the first step, the χ_M_*T* value descends to 1.78 cm^3^·K·mol^−1^ at 120 K corresponding to about 50% of the iron centres in the HS state. Further cooling reveals a second, gradual and incomplete step with a χ_M_*T* value of 0.93 cm^3^·K·mol^−1^ at 50 K; about one third of the iron centres remains in the HS state. Upon heating, a 3 K wide hysteresis is observed in the region of the first step with *T*_1/2_↑ = 127 K and *T*_1/2_↓ = 130 K. In the temperature range between 75 and 100 K first a decrease and then an increase of the χ_M_*T* product upon heating is observed. This is due to a kinetic trapping effect, often observed in this temperature region when the thermal spin transition temperature (*T*_1/2_) and the transition temperature for the thermally trapped exited spin state (*T*_TIESST_) are in close proximity [44,52–54]. In such a case the completeness of the spin crossover, in this case the second step, strongly depends on the scan rate used for the magnetic measurements. For the measurements presented in Figure 1, the settle mode was used, which corresponds to an approximate scan rate of 0.3 K·min^−1^. This allows the system to equilibrate at each temperature step where a measurement point is taken and kinetic effects can be considered to be almost irrelevant. Despite the very slow measurements, upon cooling a part of the iron centres remain trapped in the HS state. Upon slow heating they equilibrate to the LS state as long as the temperature is below the thermal spin transition temperature, which leads to the observed decrease of the χ_M_*T* product upon heating. An even slower scan rate would lead to a more complete spin transition and the disappearance of the decrease of the χ_M_*T* product upon heating while a higher scan leads to the complete disappearance of the second step. The two-step behaviour is similar to the one observed for {[FeL_eq_bpea)]·0.25MeOH}*_n_*, where the temperatures differ slightly and the second step is complete [44]. The differences due to the impact of the different solvents are also reflected in the powder diffraction patterns (Supporting Information File 1, Figure S1) in which some of the reflexes are shifted compared to the sample prepared in methanol. Sample **2** ([FeL_eq_(bpee)]*_n_*) is paramagnetic at room temperature with a χ_M_*T* value of 3.20 cm^3^·K·mol^−1^ (Supporting Information File 1, Figure S2). Upon cooling the sample remains in the HS state over the whole temperature range, as already reported for the complex synthesised from methanol [50]. Sample **3** ([FeL_eq_(bpey)]) is paramagnetic at room temperature with a χ_M_*T* value of 3.23 cm^3^·K·mol^−1^, typical for iron(II) complexes in the HS state (bottom of Figure 1). Upon cooling the χ_M_*T* value remains almost constant down to 190 K (χ_M_*T* value: 3.14 cm^3^·K·mol^−1^), where an abrupt and incomplete spin transition occurs with about 50% of the iron centres involved. The χ_M_*T* value drops to 1.73 cm^3^·K·mol^−1^ at 165 K and no further changes are observed down to 50 K (χ_M_*T* value: 1.63 cm^3^·K·mol^−1^). Upon heating up to 300 K an abrupt spin transition takes place revealing a hysteresis with a width of 10 K and *T*_1/2_↓ = 177 K and *T*_1/2_↑ = 187 K. For the sake of completeness, the complex was also synthesised from methanol yielding the same spin-crossover properties, in good agreement with the absence of solvent molecules in the crystal packing. Mössbauer spectra were collected for all three samples to verify the HS state at room temperature. The spectra (Supporting Information File 1, Figure S3) reveal one quadrupole split doublet in each case with parameters for the quadrupole splitting Δ*E*_Q_ and an isomer shift δ (Supporting Information File 1, Table S1) in the range expected for iron(II) HS complexes of this ligand type [55]. The steps and the incomplete spin crossover observed in the magnetic measurements could be due to inequivalent iron centres [56–57]. The Mössbauer spectra do not support this as no line broadening (FWHM Γ in Supporting Information File 1, Table S1) is observed and the doublet is very symmetric in each case. Thus the steps observed in the transition curve are due to the packing of the CP in the crystal and will strongly depend on the crystallinity of the material.

### Synthesis of the nanocomposite

For the CP–BCP composites [FeL_eq_(bpea)]*_n_*@BCP (**1a**–**e**), [FeL_eq_(bpee)]*_n_*@BCP (**2a**–**e**) and [FeL_eq_(bpey)]*_n_*@BCP (**3a**–**e**), the starting iron(II) complex [FeL_eq_(MeOH)_2_] and the block copolymer were dissolved in THF and refluxed for 2 h. After cooling down to room temperature, the respective bridging ligand was added and the mixture was refluxed again for 1 h. Depending on the number of additions of starting material, either the solvent is removed by cold distillation (1 cycle, samples **1a**, **2a**, **3a**) or a further cycle of addition of [FeL_eq_(MeOH)_2_] and axial ligand (simultaneously for all further cycles) followed by reflux for 1 h was performed prior to solvent removal (samples **1b**–**e**, **2b**–**e**, **3b**–**e** for 2 to 5 cycles). The resulting solids were dried in vacuo. IR spectroscopy was used to follow the formation of the coordination polymer in the BCP matrix. The corresponding spectra are given in Supporting Information File 1, Figure S4. The increasing relative intensity of the C=O stretching vibration of [FeL_eq_] clearly indicates the formation of the coordination polymer in the matrix. Elemental analysis also confirms the increasing concentration of the coordination polymer in the BCP micelle with an increasing nitrogen content.

Room-temperature Mössbauer spectra were collected of [FeL_eq_(bpea)]_n_@BCP after four and after five cycles (**1d** and **1e**) and of [FeL_eq_(bpey)]_n_@BCP after four and five cycles (**3d** and **3e**) to get a deeper insight into the sample composition. Due to the long measurement time of the very diluted (low iron content) and soft (low Lamb–Mössbauer factor) composite materials, only the more crystalline samples with a high CP amount (**d** and **e**) showing spin crossover were characterised. The corresponding spectra are given in Figure 3 (**1d** and **3e**) and in Supporting Information File 1, Figure S5. The Mössbauer parameters are summarised in Supporting Information File 1, Table S2. For the composite materials, different iron species are possible due to the coordination of the starting complex [Fe(L_eq_)] to the vinylpyridine parts of the equatorial ligand, which can be distinguished using Mössbauer spectroscopy. Sample **1d** shows two different doublets which correspond to an iron(II) HS and iron(II) LS species (75% and 25%). The LS species derives from two P4VP units coordinated to the iron centre as already shown [13,58], with the formula [Fe(L_eq_)(VP)_2_] (VP = vinyl pyridine) The HS species corresponds to the desired [Fe(L_eq_)(bpea)] unit. For sample **1e** again two doublets are observed with a similar HS/LS ratio (Supporting Information File 1 ,Table S2). The sample **3d** also shows two different iron species of which one corresponds to an iron(II) in the HS state and the other one to an iron(II) in the LS state. However, the HS/LS ratio changes to 83%:17%. For sample **3e** only one doublet is observed that can be assigned to an iron(II) HS species. It concludes that in the case of [FeL_eq_(bpey)]*_n_*@BCP the HS fraction increases with higher cycles since more or longer coordination polymer is formed in the BCP micelle, in agreement with previous observations for [FeL_eq_(bipy)]*_n_*@BCP[13,58]. In the case of [FeL_eq_(bpea)]*_n_*@BCP a different behaviour is observed that is indicative for differences in the sample composition.

### Characterisation of the nanocomposite

**Figure 2:**
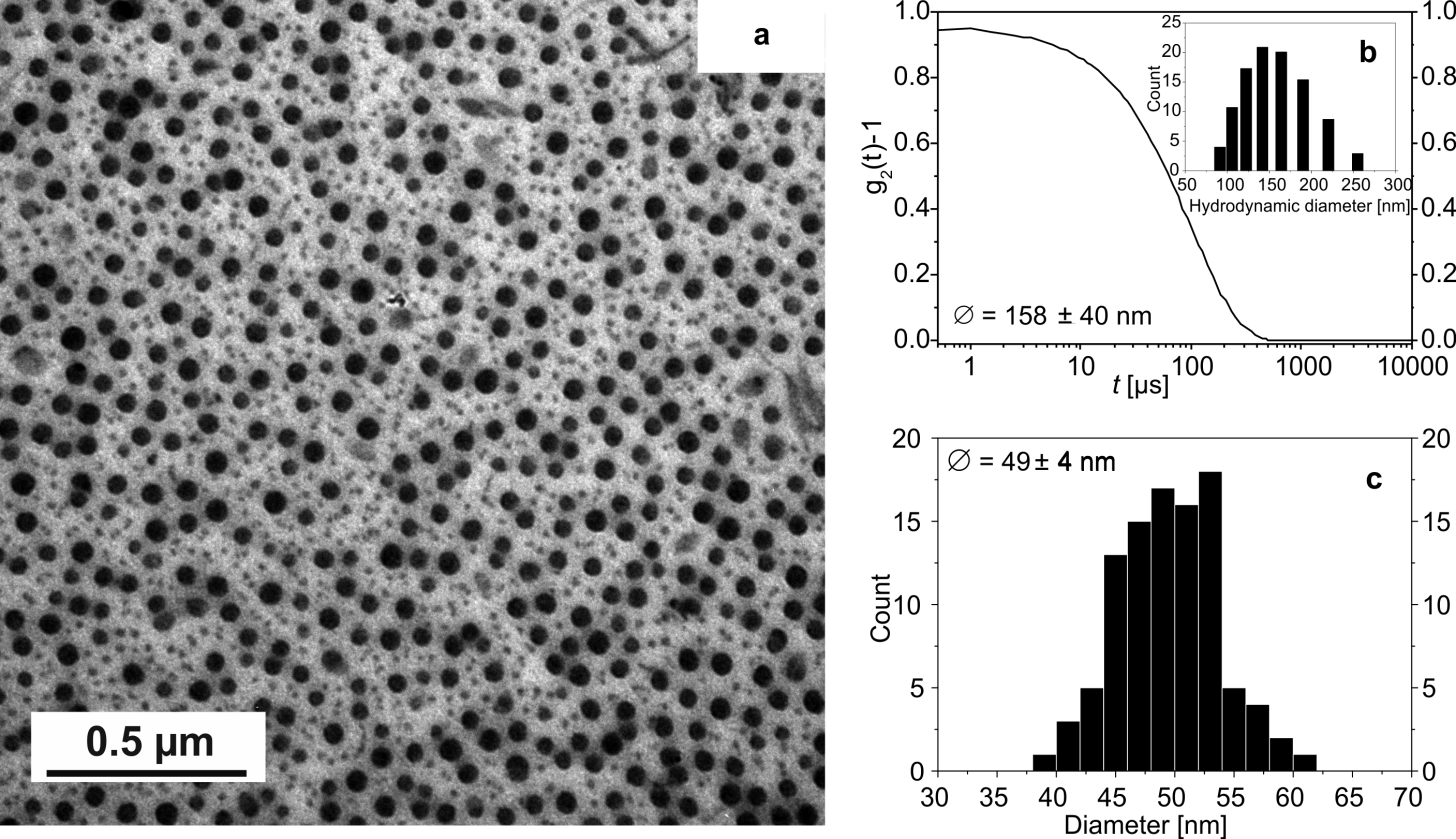
Characterisation of CP–BCP composite micelles. a) TEM picture of **3e** ([FeL_eq_(bpey)]*_n_*@BCP, five cycles) illustrating the core–shell nature of the particles. b) Autocorrelation function from dynamic light scattering of **3e** in THF (43 wt %) with size histogram. c) Size histogram of the core from the TEM picture given in panel a).

Particle sizes of the nanocomposites were determined by dynamic light scattering (DLS) in solution, transmission electron microscopy (TEM) and powder X-ray diffraction (PXRD) in the solid. The hydrodynamic diameter of the polymeric micelles loaded with the CP measured by DLS is constant within the error of the measurement throughout all measured samples with sizes around 150 nm (Supporting Information File 1, Figure S6). This is in agreement with the results reported previously for similar composite nanoparticles with 4,4′-bipyridine as bridging axial ligand [13]. In Figure 2, a TEM picture and the size distribution obtained from TEM and DLS of **3e** ([FeL_eq_(bpey)]*_n_*@BCP, five cycles) is given as typical representative of all samples. A detailed characterisation of all samples with TEM is given in Supporting Information File 1, Table S3. The TEM picture of **3e** in Figure 2a clearly reveals the formation of spherical nanoparticles with a core–shell nature. The differences in contrast of the iron-containing CP and the BCP prove that the CP nanoparticles are solely formed in the core of the nanocomposite.

The particle core diameter is significantly smaller than the hydrodynamic radius because of the polymeric nature of the BCP (solvent-swollen). Within the error of the measurement, the NP core size is in the same order of magnitude for all samples with an average size of 45 nm (Table 1), demonstrating the excellent size control by the micelles themselves. The NP core size is independent of the number of cycles and independent of the used coordination polymer clearly demonstrating the high potential of the templating effect of BCP micelles (cage effect). This in in very good agreement with our first observation on the similar system with 4,4′-biypridine as bridging ligand. It can be explained with the assumption, that the nanocomposite is very amorphous at the beginning with a low density in the core. With increasing coordination polymer concentration the crystallinity of the core and therefore its density increases, while the size does not change significantly [13].

**Table 1:** Investigation of the core size [nm] and crystallinity of the CP-BCP composite obtained from TEM. “MC” denotes the observation of microcrystals.

L_ax_	cycles				
	1 (**a**)	2 (**b**)	3 (**c**)	4 (**d**)	5 (**e**)

**bpea (1a**–**e)**	42 ± 5	46 ± 4	49 ± 4	46 ± 4	49 ± 4/MC
**bpee (2a**–**e)**	40 ± 4	46 ± 5	42 ± 4	48 ± 4/MC	47 ± 4/MC
**bpey (3a**–**e)**	48 ± 5	46 ± 4	49 ± 6	49 ± 4	49 ± 4
**bipy** [13]	52 ± 8	57 ± 8	62 ± 13	44 ± 6	49 ± 5

In order to investigate, whether the flexibility of the used bridging ligand has an impact on regioselectivity of the nanoparticle core formation, the samples were carefully analysed for the observation of microcrystals as function of the increasing CP concentration (number of cycles, e.g., [FeL_eq_(bpea)]*_n_*@BCP = **1a**–**e** for one to five cycles of addition of starting material) in the composite material. The results are summarised in Table 1.

The first microcrystals (3–6 µm) were observed for bpee as bridging ligand after four cycles of addition of starting material (**2d**), while for the more flexible bpea the first microcrystals are observed only after five cycles (**1e**, 1.5–2.0 µm). In the case of the more rigid bpey, no microcrystals are observed. This cannot solely be explained with the rigid nature of the ligand, which increases in the order bpea < bpee < bpey. One possibility to explain the observed order is to consider the stability of the complexes with regard to M–L ligand exchange with excess axial ligands and/or solvent molecules. For octahedral complexes, a weak ligand field splitting leads to the occupation of antibonding orbitals (HS complexes) and by this supports ligand exchange. A fast ligand exchange will increase the probability of the formation of microcrystals outside the BCP micelle. In this case the templating effect of the BCP micelles does not work. In agreement with this consideration, the pure HS complex [FeL_eq_(bpee)]*_n_* with the weakest ligand field splitting is the first one where microcrystals are observed, while for the spin-crossover complexes [FeL_eq_(bpea)]*_n_*, [FeL_eq_(bpey)]*_n_* and the previously investigated [FeL_eq_(bipy)]*_n_* the expected order with regard to the rigid nature of the ligand is observed. With increasing solubility of the complex (increasing flexibility of the ligand) in the solvent used for the synthesis of the nanomaterial, the probability for the formation of microcrystals outside the BCP micelles increases. In agreement with this, it was not possible to synthesise nanoparticles of the coordination polymer [FeL_eq_(bppa)]*_n_* [44], when bppa = 1,3-di(pyridin-4-yl)propane, a very flexible ligand (high solubility), is used. Syntheses were also performed in toluene to investigate the influence of the solvent on the nanoparticle synthesis. It should be pointed out that previous investigations showed that the complexes have a higher solubility in toluene compared to tetrahydrofuran. In agreement with this, first microcrystals were observed already after two cycles for all ligands. In Supporting Information File 1, Figure S7, a TEM picture of [FeL_eq_(bpea)]*_n_*@BCP after two cycles synthesised in toluene is given as typical representative. Thus the higher solubility of the coordination polymers in toluene favours the formation of microcrystals outside of the block copolymer micelle and reduces the regioselectivity.

The influence of the CP concentration on the crystallinity of the CP–BCP nanocomposite core was investigated using PXRD. In Supporting Information File 1, Figure S8, the PXRD patterns of the composite materials are compared with those of the bulk materials **1**–**3**. In all cases, the crystallinity of the particles increases with higher CP concentration, which is indicated by sharper reflexes. It should be pointed out, that in the case of the samples **3a**–**e** even after five cycles some of the prominent reflexes observed for the bulk material are missing. Either the crystallinity of the obtained NPs is still very low or a different packing compared to the bulk material is obtained.

**Figure 3:**
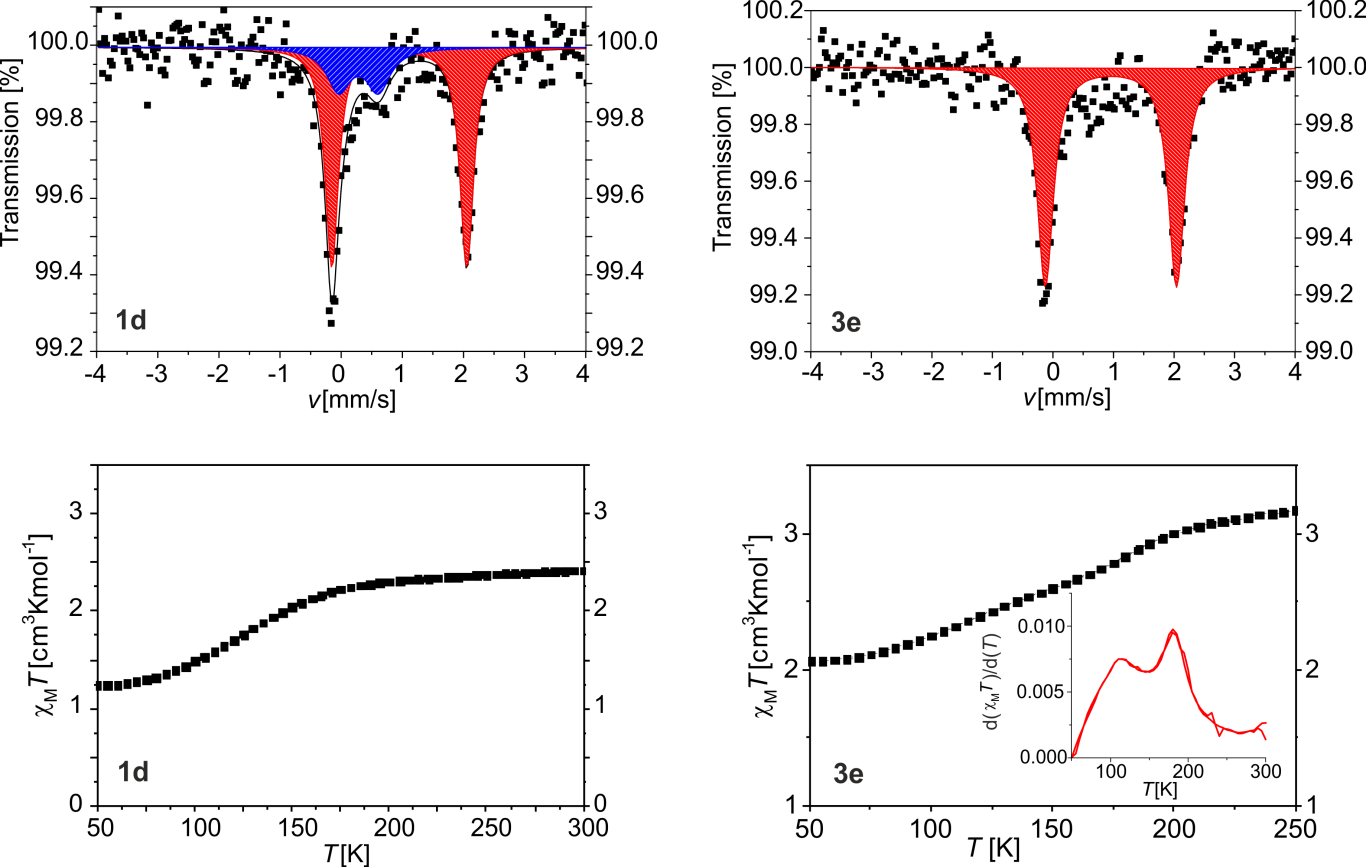
Characterisation of the magnetic properties of **1d** and **3e** Top: Mössbauer spectra of **1d** (left) and **3e** (right). Bottom: Magnetic susceptibility data displayed as χ_M_*T* vs *T* of **1d** (left) and **3e** (right). In the case of **3e** in the inset the first derivative of the χ_M_*T* vs *T* plot is given to illustrate the steps in the transition curve more clearly.

In Figure 3 (**1d** and **3e**) and Supporting Information File 1, Figure S9 (**1d,e**, **2d,e** and **3d,e**) the χ_M_*T*-versus-*T* plots of the composite materials after four and five cycles are given. Previous investigations showed, that amorphous nanoparticles of [FeL_eq_(bipy)] (1–3 cycles) showed gradual and incomplete spin crossover very different to that of the bulk material [13]. Additionally, the samples **a**–**c** are magnetically very diluted and the change in the spin state of the few SCO-active iron centres is difficult to be reliably detected. An increasing crystallinity of the nanoparticles did change the spin crossover behaviour towards that of the bulk complexes. Consequently, magnetic measurements were done for the samples **d** and **e** after four and five cycles of addition of complex in the temperature range between 50 and 300 K in the cooling and heating mode. In the case of **1d**, a gradual spin transition is observed with about 30% of the iron centres involved and *T*_1/2_ = 122 K, close to the first step of the bulk material. In contrast, sample **1e** (containing microcrystals) shows a less gradual but still incomplete spin crossover with a small hysteresis of 5 K. The χ_M_*T* value is 3.25 cm^3^·K·mol^−1^ at room temperature and decreases to 1.03 cm^3^·K·mol**^−^**^1^ at 50 K with *T*_1/2_↓ of 109 K and *T*_1/2_↑ of 114 K. Interestingly, the step in the transition curve that is present in the bulk material is not observed for sample **1e**. **3d** shows a very gradual spin crossover in the temperature range between 100 and 225 K with about 30% of the iron centres involved. This is very different to the abrupt spin transition with hysteresis of the bulk material. For sample **3e**, also a very gradual spin crossover is observed upon cooling. Two steps can be distinguished around 175 K and 110 K (see first derivative in Figure 2c). While the first step is in a similar range as the one observed for the bulk material, the second step has no relation to the spin-crossover properties of the bulk material. This is in good agreement with the results from the PXRD measurements, where pronounced differences between the diffraction pattern of the bulk CP and the nanocomposite are observed. Apparently, a different crystalline polymorph is obtained. The χ_M_*T* value is 2.07 cm^3^·K·mol^−1^ at 50 K indicating that 65% of the iron centres are still in the HS state.

## Conclusion

This work focused on the transfer of the concept for the formation of nanoparticles of coordination polymers in a block copolymer matrix. The central goal was to demonstrate that this concept of block copolymers as microreactors is not restricted to one specific coordination polymer and can easily be applied to other systems. Therefore, three coordination polymers have been chosen to be incorporated inside the block copolymer as nanoparticles. In our previous work [13] we did show that longer reaction times, higher reaction temperatures and higher amounts of CP in the BCP micelles (number of cycles) improve the crystallinity of the CP nanoparticle core while the cores size is almost constant. The improved crystallinity did change the SCO properties from gradual to abrupt with hysteresis. Here we show that the coordination polymer does not have an influence on the size of the CP–BCP composite and that the final size arises mainly from the BCP. In agreement with our previous observations, the NP size does not change significantly with increasing CP concentration in the BCP micelle. However, the formation of stable nanoparticles critically depends on the coordination polymer and the solvent used for the synthesis. The investigations reveal an interplay between two different effects: (1) The rigidity and stacking features of the bridging ligand influences the solubility of the CP and a low solubility is favourable for the selective formation of crystalline nanoparticles in the BCP micelle. (2) Weak-field ligands lead to HS complexes where anti-bonding orbitals are occupied. This supports ligand exchange and prevents the templating effect of the BCP micelle. We found that the CP–BCP composites with the most rigid ligand ([FeL_eq_(bpey)]*_n_*@BCP, **3a**–**e**) form the most stable crystalline nanoparticles that are spin-crossover active. For the HS complexes [FeL_eq_(bpee)]*_n_*@BCP, (**2a**–**e**), first microcrystals are observed after four cycles and for [FeL_eq_(bpea)]*_n_*@BCP, (**1a**–**e**) with the most flexible ligand microcrystals are observed after five cycles in THF. The differences observed for the SCO properties and the PXRD data of the bulk material **3** and the composite material **3e** are one further example for the influence of micelle formation on the crystallisation of a material [59].

## Experimental

All syntheses were performed under inert conditions using argon 5.0 (purity ≥ 99.999%) and Schlenk technique. The synthesis of all samples was repeated at least twice. Polystyrene-*b*-poly(4-vinylpyridine) (PS-P4VP, purum, *M*_W_ ≈ 150.000) was synthesised as described before [15]. 1,2-di(pyridin-4-yl)ethane) (bpea) and trans-1,2-di(pyridin-4-yl)ethene (bpee) were obtained from Sigma-Aldrich and used as received. Tetrahydrofurane (THF) p.a. and toluene were obtained from Sigma-Aldrich and degassed with argon for at least 30 min. [FeL_eq_(MeOH)_2_] was synthesized as described before [60]. The ligand bpey was synthesised according to the literature [61].

### Synthesis

The same synthesis procedures were used for all samples independent of the used L_ax_. Therefore, the general procedures are given for [FeL_eq_(bpea)]*_n_* (**1**) and the composite materials [FeL_eq_(bpea)]*_n_*@BCP (**1a**–**e**), and the specific values for [FeL_eq_(bpee)]*_n_* (**2**)/[FeL_eq_(bpey)]*_n_* (**3**) and the composite materials [FeL_eq_(bpee)]*_n_*@BCP (**2a**–**e**)/[FeL_eq_(bpey)]*_n_*@BCP (**3a**–**e**) are given in brackets. The synthesis of the composite materials in toluene was done using the same procedures and amounts as described for THF. Due to the observation of microcrystals at a very early stage, the products were not characterized further.

**1** (**2**/**3**): 200 mg (0.45 mmol) [FeL_eq_(MeOH)_2_] and 206 mg (204 mg/202 mg) (1.125 mmol, 2.5 equiv) bpea (bpee/bpey) were dissolved in 20 mL THF in a 50 mL flask. The solution was refluxed for 1 h. After cool-down to room temperature, the solution was let for crystallisation overnight. The solid was filtered, washed with THF once and dried in vacuo to yield a brown (dark violet) powder. Elemental analysis, Anal. calcd for C_30_H_30_N_4_O_4_Fe (**1**): C, 63.61; H, 5.34; N, 9.89; found: C, 62.91; H, 5.19; N, 9.22; (Anal. calcd for C_30_H_28_N_4_O_4_Fe (**2**): C, 63.84; H, 5.00; N, 9.93; found: C, 63.15; H, 6.05; N, 9.18/Anal. calcd for C_30_H_26_N_4_O_4_Fe (**3**): C, 64.07; H, 4.66; N, 9.96; found: C, 63.63; H, 4.77; N, 9.25).

**1a**, one cycle (**2a**/**3a**): 50 mg (0.33 µmol) PS-*b*-P4VP and 6.7 mg (15 µmol) [FeL_eq_(MeOH)_2_] were dissolved in 20 mL THF in a 50 mL flask. The solution was refluxed for 2 h. After, 6.9 mg (6.8 mg/6.8 mg) (37.5 µmol, 2.5 equiv) bpea (bpee/bpey) was added and refluxed again for 1 h. The solution was cooled down to room temperature and the solvent was removed via cold distillation to yield a brown, polymer-like solid. Elemental analysis, found: C, 64.96; H, 7.44; N, 2.82; (C, 71.23; H, 7.24; N, 3.10/C, 59.99; H, 7.46; N, 2.48).

**1b**, two cycles (**2b**/**3b**): The synthesis for one cycle was repeated. Prior to solvent removal, 6.7 mg (15 µmol) [FeL_eq_(MeOH)_2_] and 6.9 mg (6.8 mg/6.8 mg) (37.5 µmol, 2.5 equiv) bpea (bpee/bpey) were added for a new cycle and refluxed for another hour. The solvent was removed via cold distillation to yield a dark brown, polymer-like solid. Elemental analysis, found: C, 61.98; H, 7.35; N, 3.38; (C, 59.75; H, 7.43; N, 3.37/C, 57.18; H, 7.42; N, 3.05).

**1c**, three cycles (**2c**/**3c**): The synthesis for two cycles was repeated and one more cycle was carried out. 6.7 mg (15 µmol) [FeL_eq_(MeOH)_2_] and 6.9 mg (6.8 mg/6.8 mg) (37.5 µmol, 2.5 equiv) bpea (bpee/bpey) were added and refluxed for another hour before the solvent was removed via cold distillation to yield a dark brown, polymer-like solid. Elemental analysis, found: C, 69.43; H, 7.30; N, 5.00 (C, 63.08; H, 7.21; N, 3.71/C, 70.94; H, 6.67; N, 4.88).

**1d**, four cycles (**2d**/**3d**): The synthesis for three cycles was repeated and one more cycle was run. 6.7 mg (15 µmol) [FeL_eq_(MeOH)_2_] and 6.9 mg (6.8 mg/6.8 mg) (37.5 µmol, 2.5 equiv) bpea (bpee/bpey) were added and refluxed for another hour before the solvent was removed via cold distillation to yield a dark brown, polymer-like solid. Elemental analysis, found: C, 68.18; H, 6.55; N, 5.64 (C, 71.09; H, 6.79; N, 5.90/C, 68.04; H, 6.18; N, 5.48).

**1e**, five cycles (**2e**/**3e**): The synthesis for four cycles was repeated and one more cycle was run. 6.7 mg (15 µmol) [FeL_eq_(MeOH)_2_] and 6.9 mg (6.8 mg/6.8 mg) (37.5 µmol, 2.5 equiv) bpea (bpee/bpey) were added and refluxed for another hour before the solvent was removed via cold distillation to yield a dark brown, polymer-like solid. Elemental analysis, found: C, 68.09; H, 6.97; N, 5.86; (C, 68.12; H, 6.63; N, 6.09/C, 65.92; H, 6.04; N, 5.70).

The colour of the samples became darker with increasing cycles due to the higher amount of iron inside the samples. The increasing nitrogen content in the elemental analysis from **a**–**e** also confirms the increasing amount of coordination polymer in the samples.

### Characterisation methods

**Transmission electron microscopy:** Transmission electron microscopy was carried out at a Zeiss CEM902 electron microscope (Zeiss, Oberkochen, Germany). Samples were dispersed in toluene applying vortex. The solution was dropped on a copper grid (mesh 200, Science Services, Munich). Electron acceleration voltage was set to 80 kV. Micrographs were taken with a MegaView III/iTEM image acquiring and processing system from Olympus Soft Imaging Systems (OSIS, Münster, Germany) and an Orius 830 SC200W/DigitalMicrograph system from Gatan (Munich, Germany). Particles size measurements were done with “ImageJ” image processing software by Wayne Rasband (National Institutes of Health, USA).

**Elemental analysis:** Carbon, nitrogen and hydrogen content was measured using a Vario EL III with acetanilide as standard. The samples were placed in tin boats and measured at least twice. The average of the measurements was used.

**Infrared spectroscopy measurements:** Transmission infrared spectra were collected using a Perkin Elmer Spectrum 100 FTIR (ATR). The samples were measured directly as solids.

**Magnetic measurements:** Magnetic susceptibility measurements were performed with a Quantum Design MPMS-XL-5 SQUID magnetometer. Field strength of 3 T was applied and a temperature range of 50–300 K was used to determine the temperature dependency of the magnetism and the spin-crossover behaviour. Settle mode was used in all measurements with a cooling and heating rate of 5 K/min. The samples were prepared in gelatine capsules placed in a plastic straw. The measured values were corrected for the diamagnetism of the sample holder, the polymer matrix (measured values) and the ligand (tabulated Pascal constants).

**Dynamic light scattering:** The samples were measured using a Malvern Instruments Zetasizer Nano ZS90 in glass cuvettes from Carl Roth GmbH + Co. KG at 25 °C. One measurement consisted of three consecutive runs.

**Mössbauer spectroscopy:** ^57^Fe Mössbauer spectra were recorded in transmission geometry under constant acceleration using a conventional Mössbauer spectrometer with a 50 mCi ^57^Co(Rh) source. The samples were sealed in the sample holder in an argon atmosphere. The spectra were fitted using Recoil 1.05 Mössbauer Analysis Software [62]. The isomer shift values are given with respect to α-Fe as reference at room temperature. At present, only measurements at room temperature are possible with the instrumental setup.

**Powder X-ray diffraction:** Powder X-ray diffraction data for all samples were collected at a STOE StadiP X-Ray diffractometer in transmission geometry in a 2θ range of 5–30°. Samples **1**, **2** and **3** were placed in capillaries and composite samples **1a**–**3e** were placed on flat surfaces. Cu Kα_1_ radiation was used for the measurement and the radiation was detected with a Mythen 1K detector.

## Supporting Information

In the Supporting Information the characterization of the bulk complexes (PXRD, magnetism, Mössbauer spectra and Mössbauer parameter), the full characterization of the composite materials **1a**–**e, 2a**–**e** and **3a**–**e** (IR spectra, DLS, PXRD, Mössbauer spectra and Mössbauer parameters of **1d**, **1e**, **3d** and **3e**, TEM pictures and magnetic measurements of **1d**, **1e**, **2d**, **2e**, **3d** and **3e**) and a TEM picture of the composite material synthesised from toluene are given.

File 1 Additional experimental data.
