# Supplementary material for: Synthesis of [Fe(Leq)(Lax)]n coordination polymer nanoparticles using blockcopolymer micelles
Source: Beilstein J Nanotechnol. 2017 Jun 26;8:1318–27. doi: 10.3762/bjnano.8.133 (PMC5496571; doi:10.3762/bjnano.8.133)
Supplement: File 1 — Additional experimental data. [file Beilstein_J_Nanotechnol-08-1318-s001.pdf]

Supporting Information

for

Synthesis of $[Fe(L_{eq})(L_{ax})]_n$ coordination polymer nanoparticles using blockcopolymer micelles

Christoph Göbel[1], Ottokar Klimm[1], Florian Puchtler[2], Sabine Rosenfeldt[3], Stephan Förster[3] and Birgit Weber*[1]

Address: [1]Inorganic Chemistry II, University of Bayreuth, Universitätsstr. 30, 95440 Bayreuth, Germany; [2]Inorganic Chemistry I, University of Bayreuth, Universitätsstr. 30, 95440 Bayreuth, Germany and [3]Physical Chemistry I and Bavarian Polymer Institute, University of Bayreuth, Universitätsstr. 30, 95440 Bayreuth, Germany

Email: Birgit Weber* - weber@uni-bayreuth.de

* Corresponding author

Additional experimental data

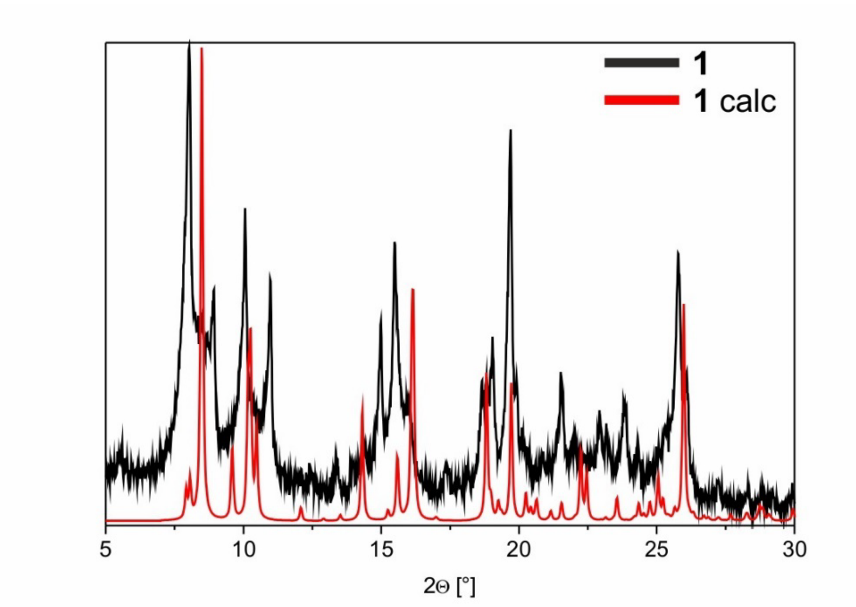

Figure S1: Comparison of the powder X-ray diffraction pattern of $[Fe(L_{eq})(bpea)]_n$ (**1**) and $\{[Fe(L_{eq})(bpea)]\cdot 0.25\ MeOH\}_n$ (synthesised in methanol, calculated from single crystal data) [1].

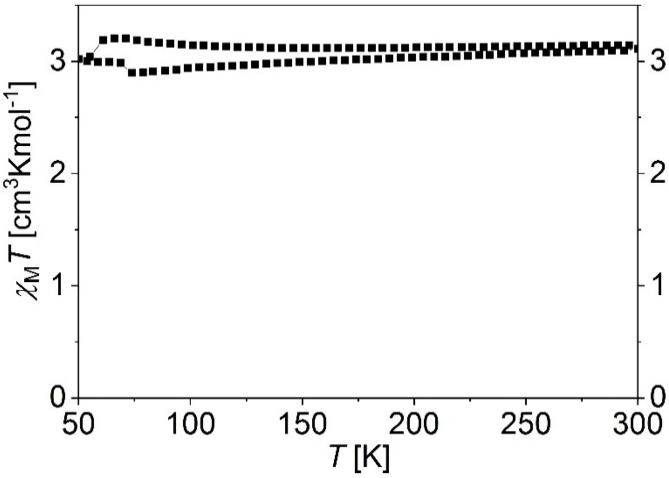

Figure S2: Plot of the $\chi_M T$ product versus temperature for **2**.

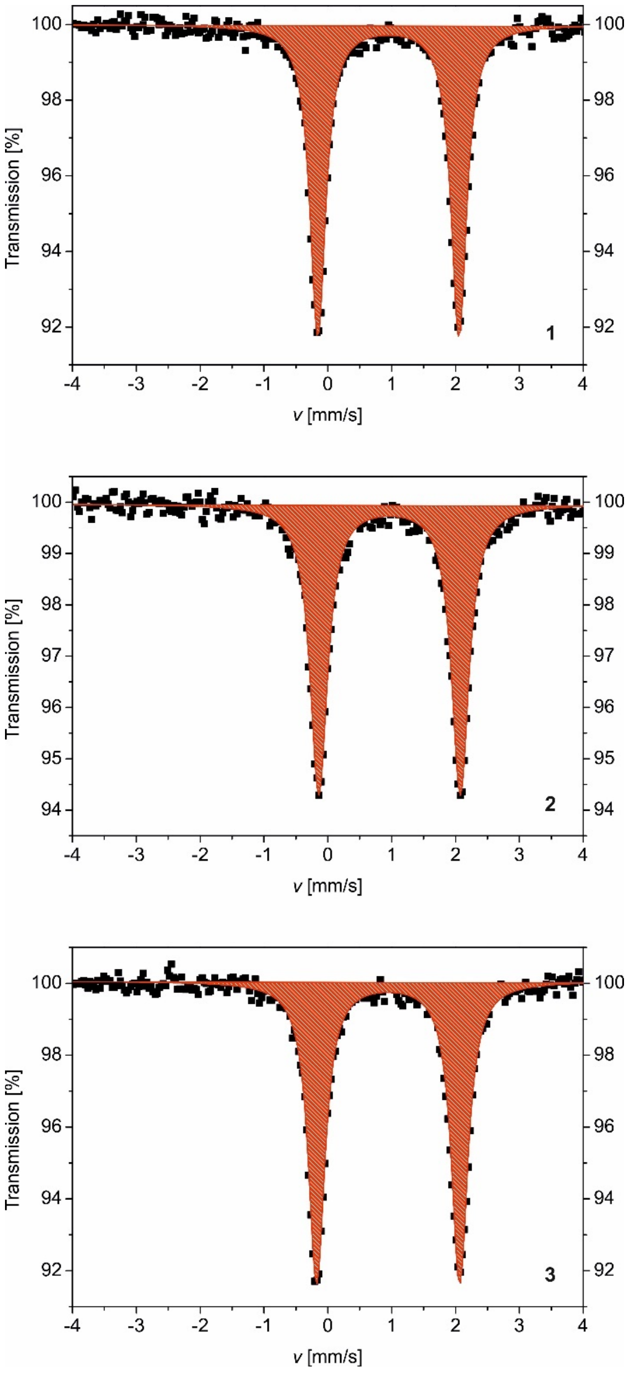

Figure S3: Mössbauer spectra of **1** (top), **2** (centre) and **3** (bottom). In each case one single doublet is observed with Mössbauer parameters (Table S1) characteristic for an iron(II) HS complex.

Table S1: Mössbauer parameters of the samples **1**, **2** and **3**.

sample	site	δ [mm/s]	ΔE_Q [mm/s]	Γ [mm/s]	Area [%]
**1**	Fe(II) HS	0.947(3)	2.210(6)	0.147(5)	100
**2**	Fe(II) HS	0.966(4)	2.216(7)	0.164(6)	100
**3**	Fe(II) HS	0.944(3)	2.240(6)	0.156(5)	100

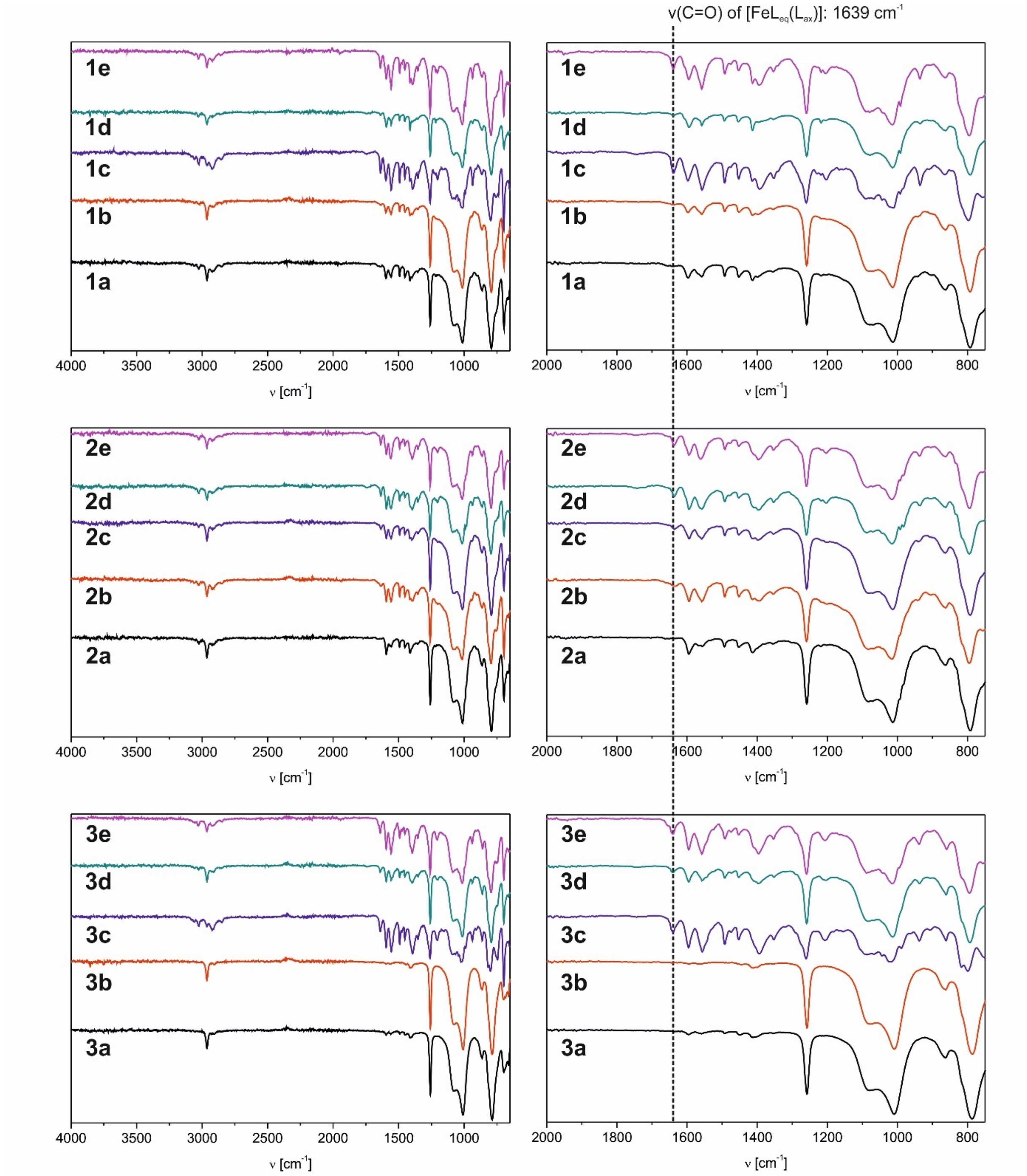

Figure S4: IR spectra of **1a**–**e** (top left), **2a**–**e** (centre left) and **3a**–**e** (bottom left) and the relevant area between 2000 and 750 cm^{-1} to show the C=O vibration band the samples **1a**–**e** (top right), **2a**–**e** (centre right) and **3a**–**e** (bottom right).

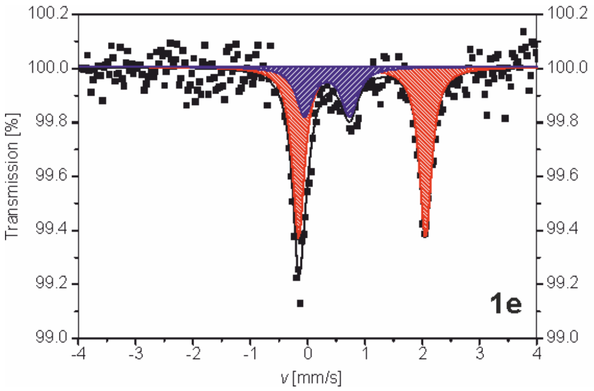

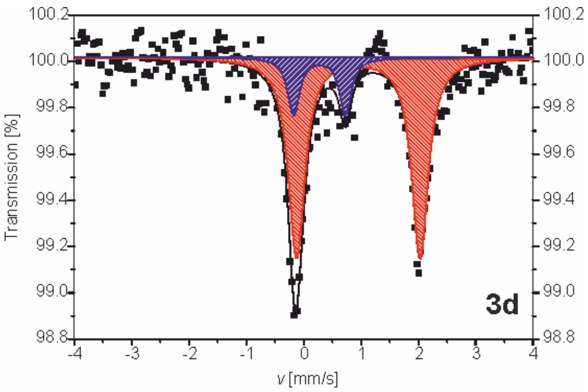

Figure S5: Mössbauer spectra of **1e** (left) and **3d** (right). The red doublet corresponds to and iron(II) HS species and the blue doublet corresponds to an iron(II) LS species. The Mössbauer parameters are given in Table S2.

Table S2: Mössbauer parameters of the samples **1d**, **1e**, **3d** and **3e**.

sample	site	δ [mm/s]	ΔE_Q [mm/s]	Γ [mm/s]	Area [%]
**1d**	Fe(II) LS	0.28(11)	0.65(18)	0.24(14)	26(9)
	Fe(II) HS	0.951(14)	2.21(3)	0.132(19)	74(9)
**1e**	Fe(II) LS	0.34(6)	0.79(12)	0.17(8)	28(9)
	Fe(II) HS	0.951(14)	2.21(3)	0.12(2)	72(9)
**3d**	Fe(II) LS	0.28(6)	0.90(12)	0.12(6)	17(6)
	Fe(II) HS	0.95(2)	2.15(4)	0.17(2)	83(9)
**3e**	Fe(II) HS	0.958(12)	2.17(2)	0.161(18)	100

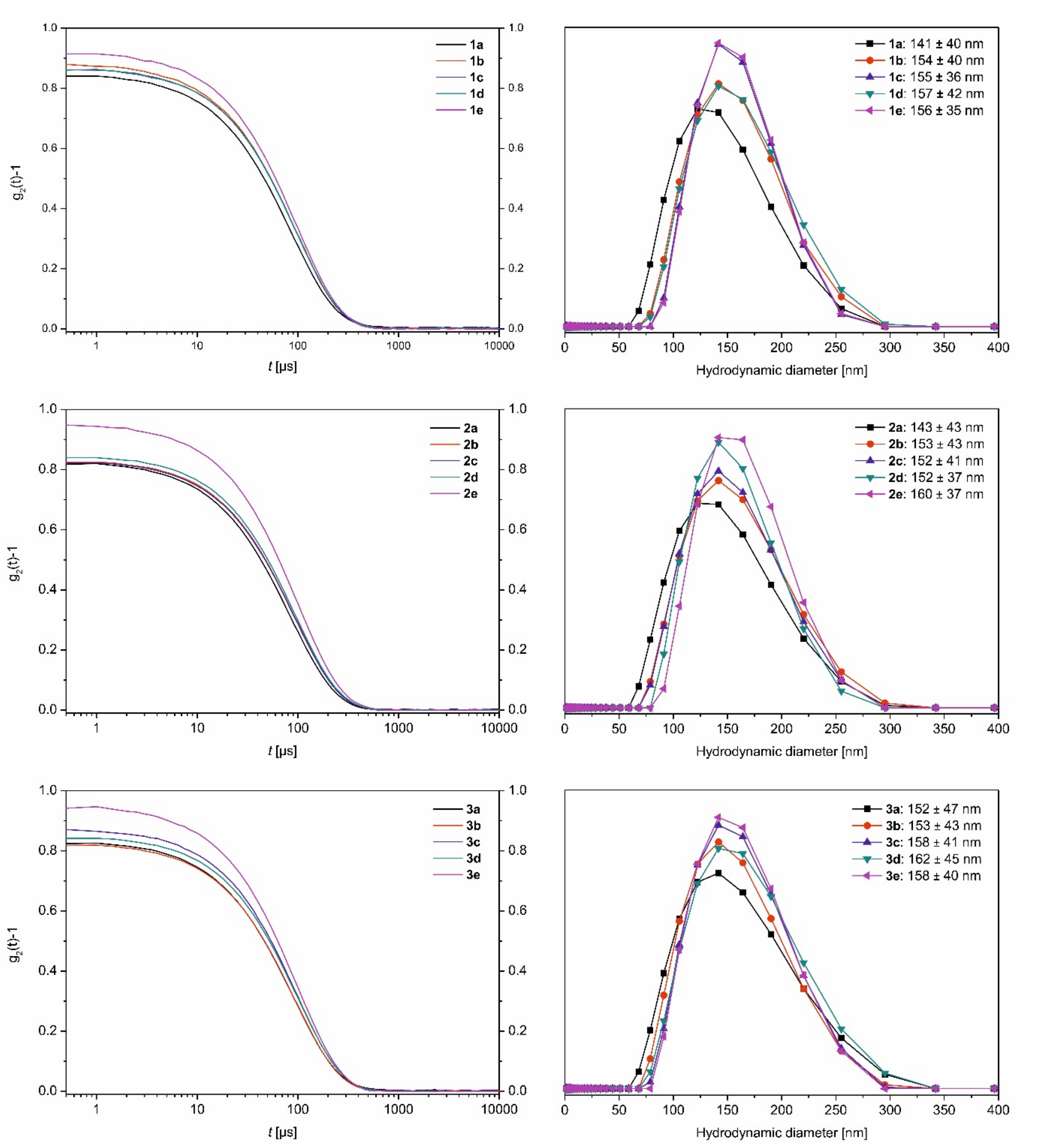

Figure S6: DLS measurement of the nanocomposites in THF, 43 wt %. Correlation functions of **1a**–**e** (top left), **2a**–**e** (centre left) and **3a**–**e** (bottom left) and the resulting hydrodynamic diameter of the polymeric micelles in THF of **1a**–**e** (top right), **2a**–**e** (centre right) and **3a**–**e** (bottom right).

Table S3: Summarised characterisation for the different composite samples (**1a**–**3e**). An exemplary TEM picture and the size distribution are given for TEM measurements. The particle sizes are given in the pictures.

sample	Reaction conditions	TEM nanoparticles, TEM size distribution, TEM microcrystals (if any) and Mössbauer spectra (if any).
**1a**	1 cycle, THF, reflux	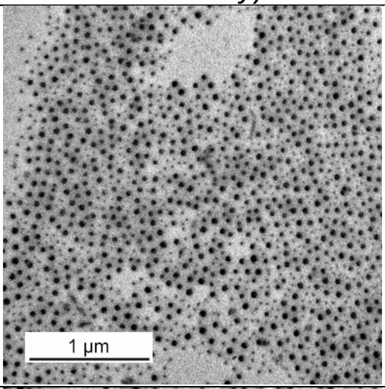 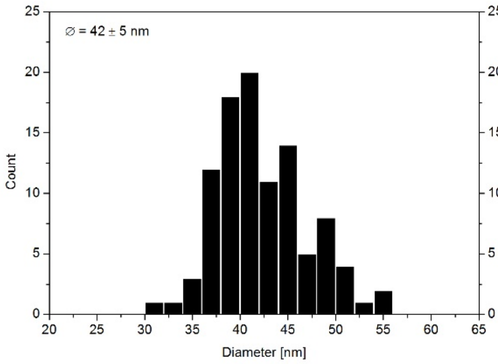
**1b**	2 cycles, THF, reflux	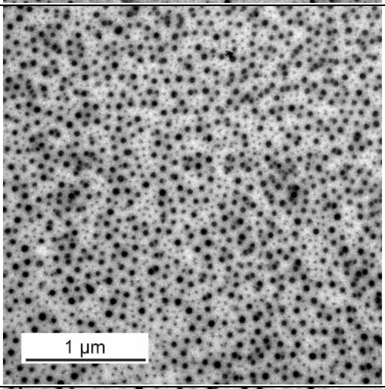 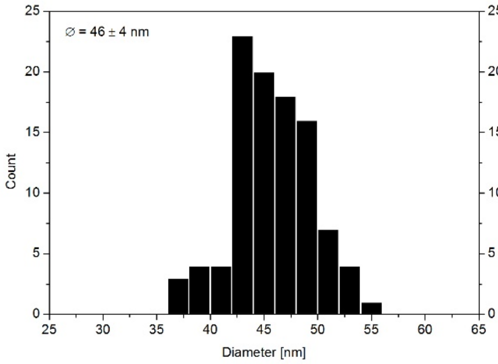
**1c**	3 cycles, THF, reflux	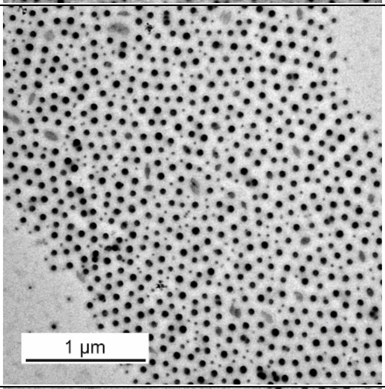 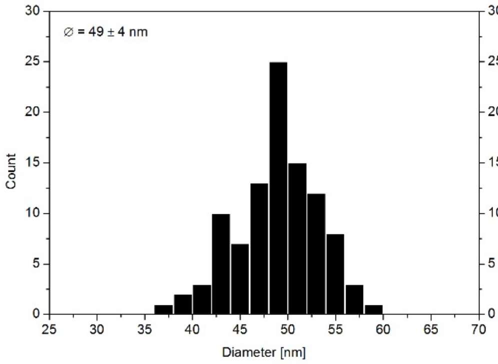
**1d**	4 cycles, THF, reflux	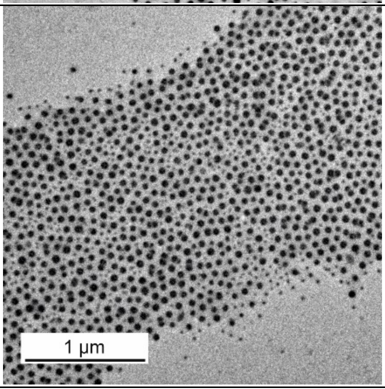 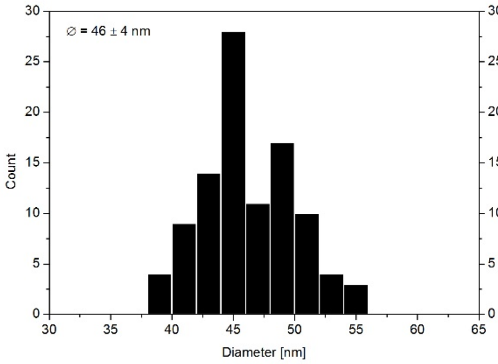

<table>
<tr><td></td><td></td><td>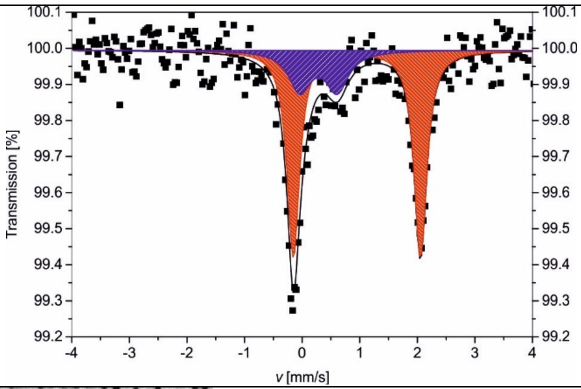</td></tr>
<tr><td>1e</td><td>5 cycles, THF, reflux</td><td>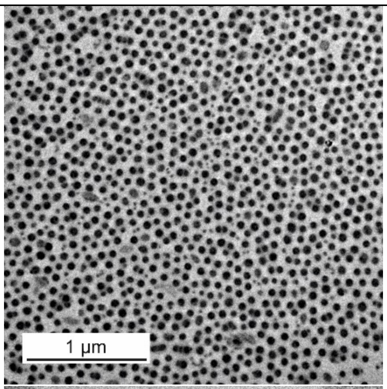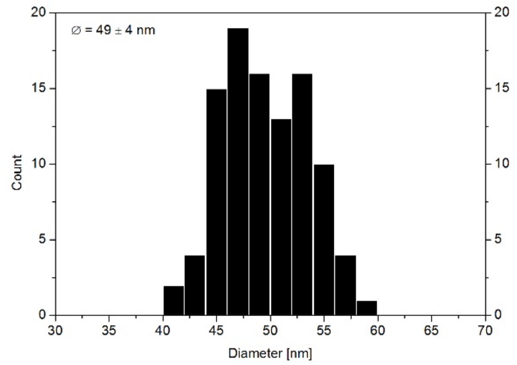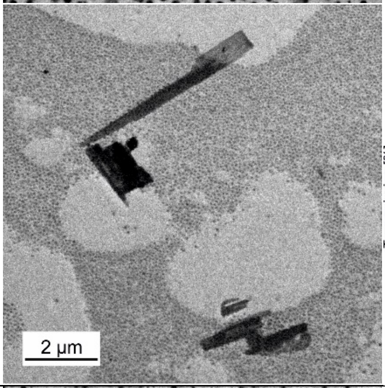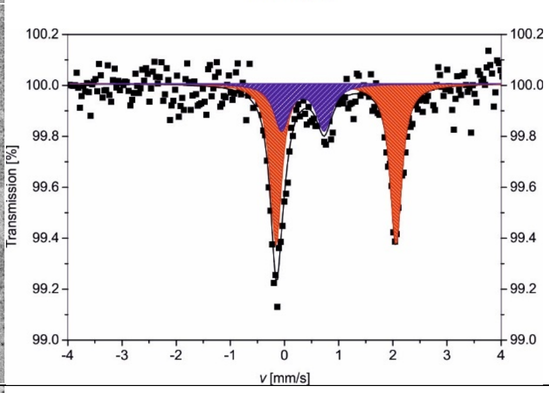</td></tr>
<tr><td>2a</td><td>1 cycle, THF, reflux</td><td>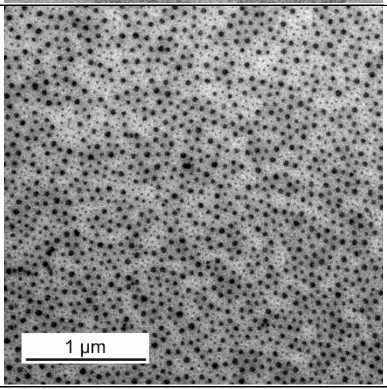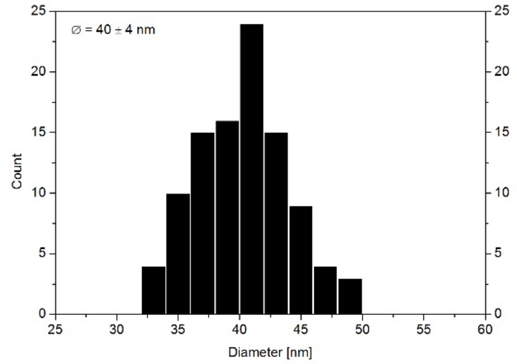</td></tr>
</table>

<table>
<tr><td>2b</td><td>2 cycles,
THF,
reflux</td><td>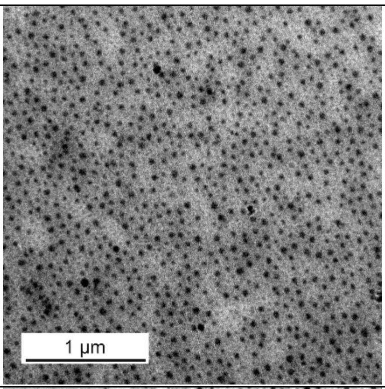

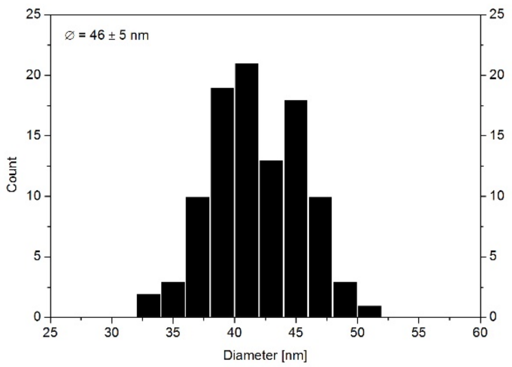
</td></tr>
<tr><td>2c</td><td>3 cycles,
THF,
reflux</td><td>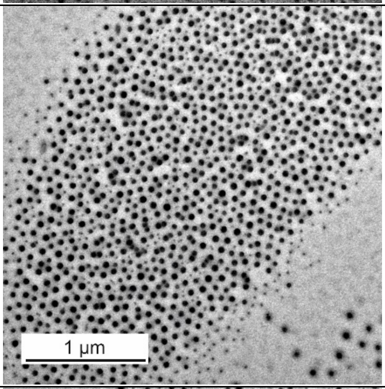

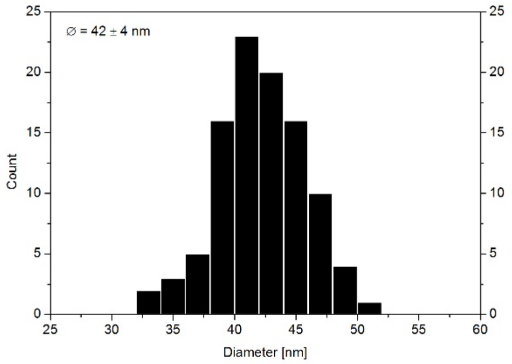
</td></tr>
<tr><td>2d</td><td>4 cycles,
THF,
reflux</td><td>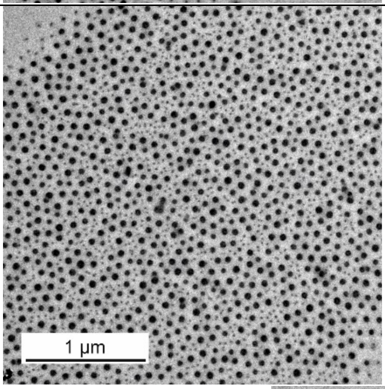

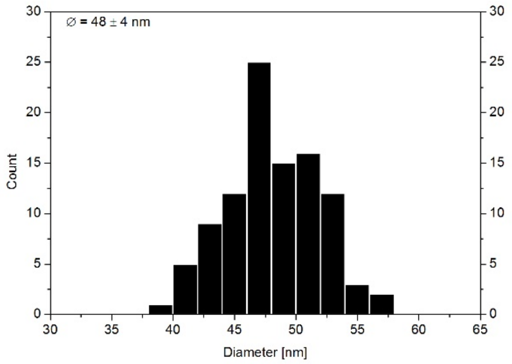

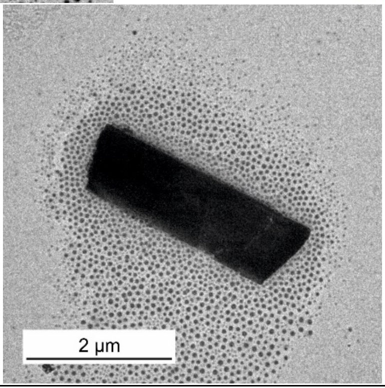
</td></tr>
</table>

**2e**	5 cycles, THF, reflux	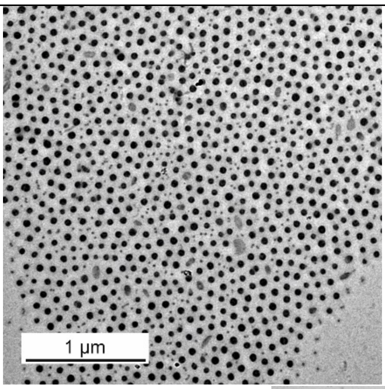 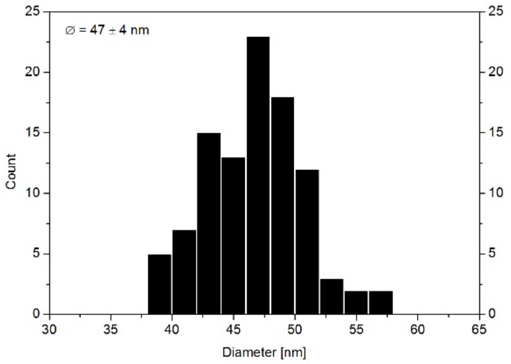 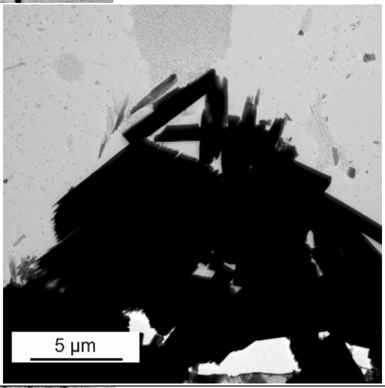
**3a**	1 cycle, THF, reflux	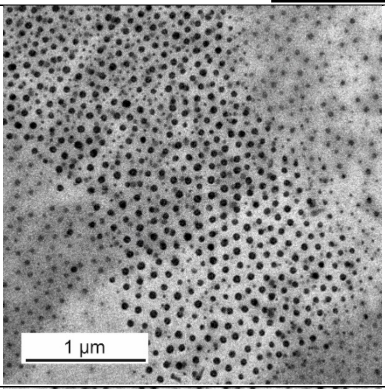 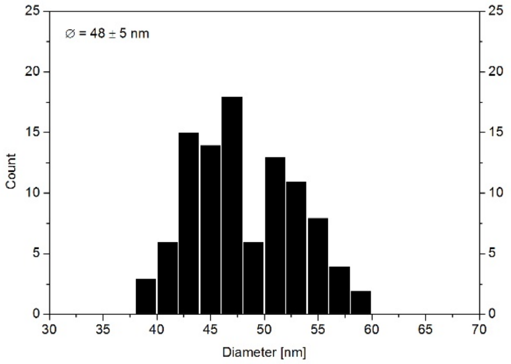
**3b**	2 cycles, THF, reflux	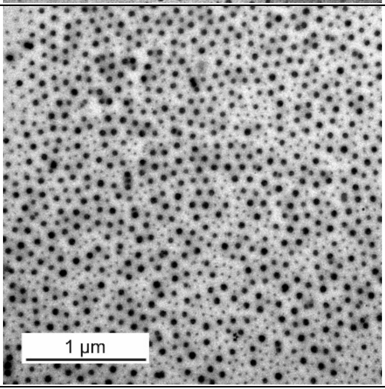 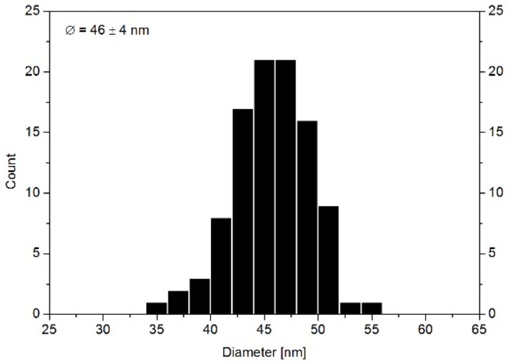

**3c**	3 cycles, THF, reflux	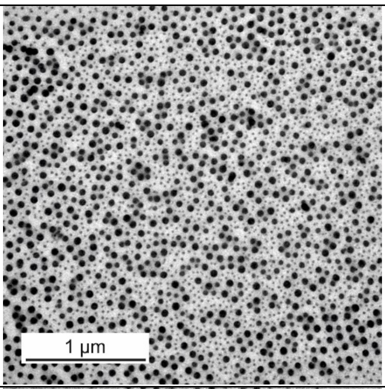 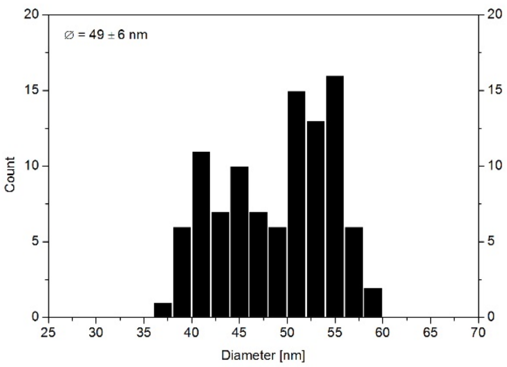
**3d**	4 cycles, THF, reflux	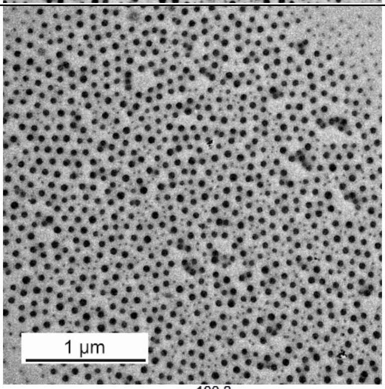 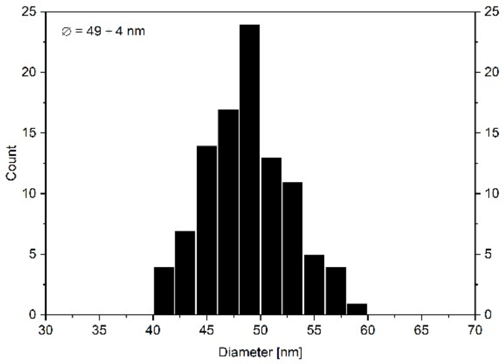 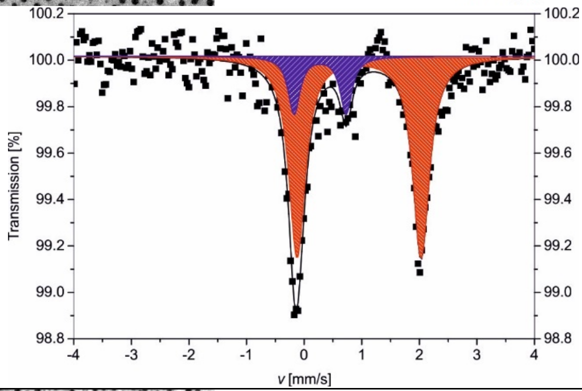
**3e**	5 cycles, THF, reflux	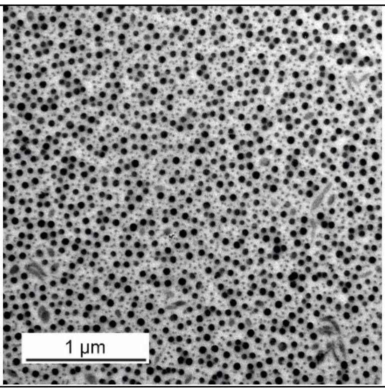 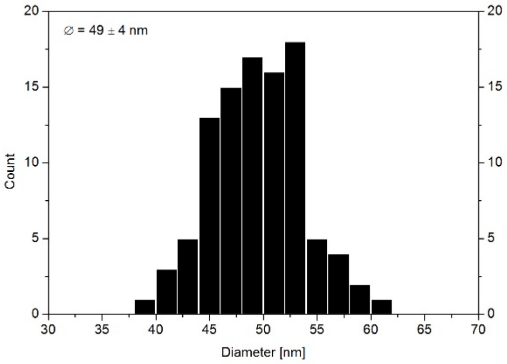

		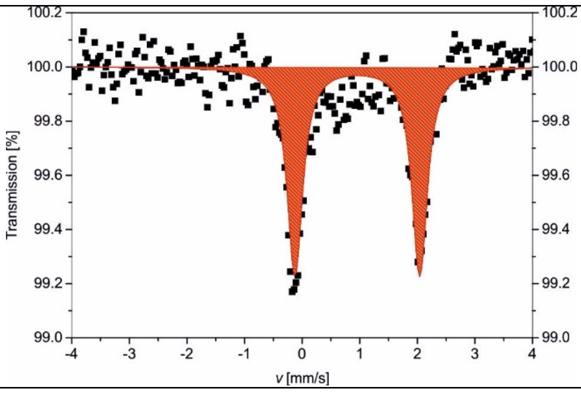100.2<br>100.0<br>99.8<br>99.6<br>99.4<br>99.2<br>99.0<br>Transmission [%]<br>-4<br>-3<br>-2<br>-1<br>0<br>1<br>2<br>3<br>4<br>v [mm/s]

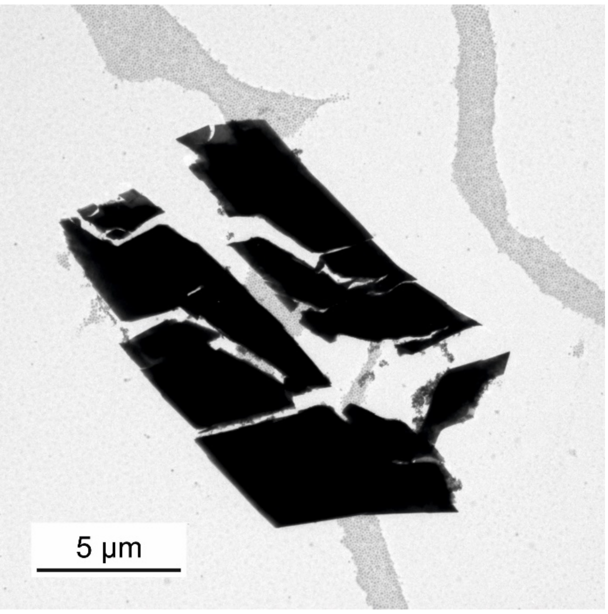

Figure S7: Exemplary TEM picture of $[FeL_{eq}(bpea)]_n$@BCP after two cycles synthesised in toluene to show microcrystals of the coordination polymer.

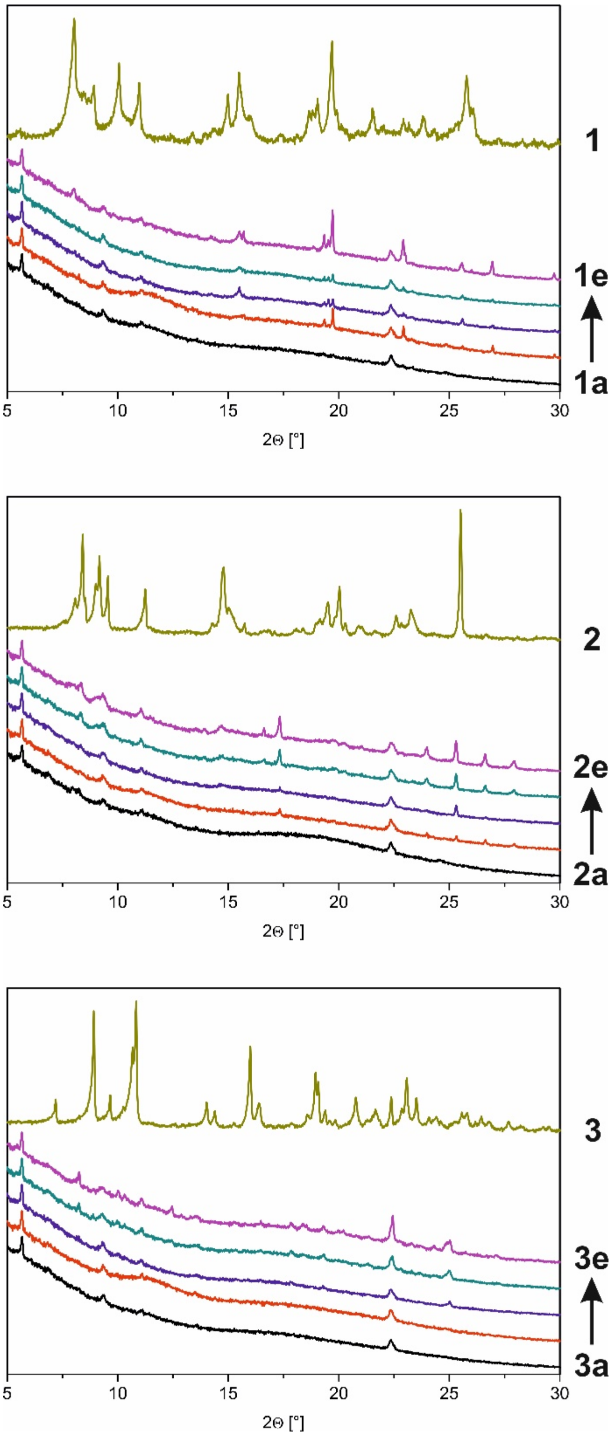

Figure S8: Powder X-ray diffraction pattern of **1** and **1a**–**e** (top), **2** and **2a**–**e** (centre) and **3** and **3a**–**e** (bottom).

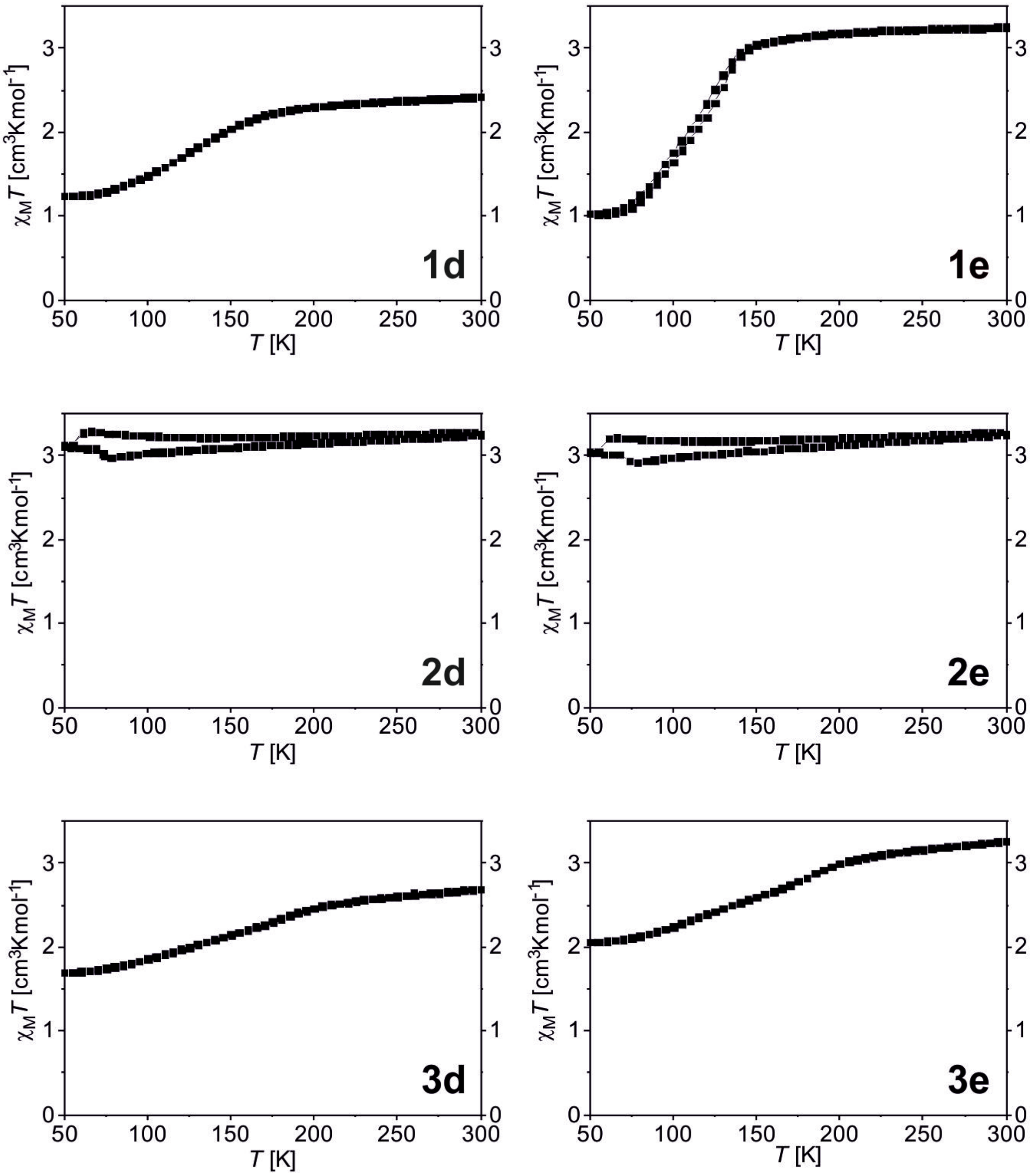

Figure S9: $\chi_M T$ vs T plots of the samples **1d** (top left), **1e** (top right), **3d** (bottom left) and **3e** (bottom right). The results for the samples **2d** (centre left) to **2e** (centre right) are identical to the bulk material [FeL_{eq}(bpee)]$_n$; the $\chi_M T$ product is constant in the temperature region investigated and no indication for spin crossover is observed. This is not surprising, as relatively large microcrystals are observed.

References

1. Bauer, W.; Scherer, W.; Altmannshofer, S.; Weber, B. *Eur. J. Inorg. Chem.* **2011,** *2011,* 2803–2818. doi:10.1002/ejic.201001363
